# Effect of Antibiotics With Anaerobic Coverage on Graft‐Versus‐Host Disease in Patients Undergoing Allogeneic Hematopoietic Stem Cell Transplantation: A Systematic Review and Meta‐Analysis

**DOI:** 10.1111/tid.70049

**Published:** 2025-04-29

**Authors:** Hiroshi Ito, Yui Okamura, Yuna Tomura, Jura Oshida, Minori Fujita, Daiki Kobayashi

**Affiliations:** ^1^ Division of General Internal Medicine, Department of Internal Medicine Tokyo Medical University Ibaraki Medical Center Ibaraki Japan; ^2^ College of Medicine, School of Medicine and Health Sciences University of Tsukuba Tsukuba Japan; ^3^ Library, Tokyo Medical University Tokyo Japan

**Keywords:** allogeneic hematopoietic stem cell transplantation, anaerobic coverage, antibiotics, graft‐versus‐host disease

## Abstract

**Background:**

Broad‐spectrum antibiotics are standard for febrile neutropenia (FN) in allogeneic hematopoietic stem cell transplantation (HSCT) but may disrupt gut microbiota, increasing the risk of graft‐versus‐host disease (GVHD). However, current evidence on the effects of anaerobic versus limited anaerobic antibiotic coverage on GVHD‐related outcomes remains inconclusive.

**Methods:**

We systematically searched for studies assessing overall survival, acute GVHD incidence, and GVHD‐related mortality in patients with allogeneic HSCT receiving antibiotics with anaerobic versus limited anaerobic coverage. A random‐effects meta‐analysis calculated risk ratios (RRs) and 95% confidence intervals (CIs) after assessing bias risk.

**Results:**

Six of the 323 screened studies met the inclusion criteria, encompassing 2169 patients: five studies included adult populations, and one included a pediatric population. Meta‐analysis revealed no significant difference in 1‐year overall survival between the anaerobic and the limited anaerobic coverage groups (RR: 1.01; 95% CI: 0.92–1.12). Acute GVHD incidence was significantly higher in the anaerobic coverage group than in the limited anaerobic coverage group (RR: 1.33; 95% CI: 1.17–1.51). GVHD‐related mortality tended to be higher in the anaerobic coverage group than in the limited coverage group (RR: 1.65; 95% CI: 0.94–2.91). Of the six studies, three had a high risk of bias. Moderate heterogeneity was observed between citations regarding GVHD‐related mortality (*I* ^2^ = 63%).

**Conclusion:**

Antibiotics with anaerobic coverage appear to increase acute GVHD incidence in patients who received an allogeneic HSCT compared to antibiotics with limited anaerobic coverage. However, the strength of this conclusion is limited by the quality of available evidence. Further well‐designed research is necessary to clarify the impact of anaerobic antibiotic coverage on GVHD‐related outcomes.

## Graphical abstract

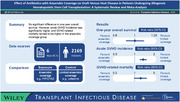

**Abbreviations**
CI: confidence interval
FN: febrile neutropenia
GVHD: graft‐versus‐host disease
HSCT: hematopoietic stem cell transplantation
PRISMA: Preferred Reporting Items for Systematic Reviews and Meta‐Analyses
RCT: randomized controlled trial
ROBINS‐I: Risk of Bias in Non‐Randomized Studies of Interventions
RR: risk ratio

## 1 Introduction

Patients undergoing allogeneic hematopoietic stem cell transplantation (HSCT) face a high risk of febrile neutropenia (FN), with incidence rates exceeding 40% [1, 2]. Given FN's potential for high mortality if sepsis develops [3], prophylactic antibiotics are administered before and after HSCT for selected patients, with antipseudomonal agents recommended. For example, the Infectious Diseases Society of America recommends the use of prophylactic antibiotics when the neutrophil count remains ≤ 100/µL for more than 7 days [4], while the National Comprehensive Cancer Network recommends prophylactic antibiotic administration when the neutrophil count remains ≤ 500/µL (or ≤ 1000/µL and a predicted decline to ≤ 500/µL over the next 48 h) for more than 7 days [5], with fluoroquinolones such as levofloxacin being commonly used until the neutrophil count recovery. On the other hand, for the treatment of patients who develop FN, intravenous antibiotics such as cefepime, piperacillin‐tazobactam, and carbapenems or oral fluoroquinolones are used as empirical therapy, depending on the level of risk [4, 5].

Graft‐versus‐host disease (GVHD) occurs in 40%–60% of patients who undergo allogeneic HSCT [6, 7], with antibiotic‐induced intestinal microbiota disruption implicated in its pathogenesis [8, 9]. A systematic review and meta‐analysis linked systemic antibiotic use to increased risk of acute GVHD [10]. Reduced gut microbiota diversity has also been associated with GVHD development [11, 12]. In a study of 31 patients who underwent HSCT, those with gastrointestinal GVHD exhibited a predominance of *Enterococcus* species in stool samples compared to those without GVHD [13]. Of note, the predominance of *Enterococcus* species in the gut microbiota can be driven by the weakening of colonization resistance due to the depletion of other intestinal bacteria, primarily anaerobes [14].

In this context, broad‐spectrum antibiotics with intestinal anaerobic coverage (e.g., piperacillin‐tazobactam, carbapenems) may elevate the risk of acute GVHD and GVHD‐related mortality in patients who are post‐HSCT compared to agents with limited anaerobic coverage (e.g., cefepime, aztreonam). However, existing studies report inconsistent findings, leaving the evidence inconclusive. To address this gap, we conducted a systematic review and meta‐analysis to evaluate whether antibiotics with anaerobic antibiotic coverage correlate with increased GVHD‐related risk.

## 2 Methods

This systematic review and meta‐analysis was registered in the University Hospital Medical Information Network Clinical Trials Registry (UMIN000055758) and adhered to the Preferred Reporting Items for Systematic Reviews and Meta‐Analyses (PRISMA) guidelines (Table S1).

### 2.1 Study Search and Selection

We systematically searched PubMed, Web of Science, and the Cochrane Library for studies published through December 31, 2024, that evaluated mortality in patients receiving antibiotics after allogeneic HSCT, comparing those with anaerobic versus limited anaerobic coverage. Antibiotics with anaerobic coverage included penicillins with β‐lactamase inhibitors, cephamycins, carbapenems, clindamycin, metronidazole, moxifloxacin, chloramphenicol, and tigecycline [15]. Limited anaerobic coverage included all other antibiotics, such as cephalosporins (excluding cephamycins), aztreonam, and fluoroquinolones (except moxifloxacin). The primary endpoint was overall survival, while acute GVHD incidence and GVHD‐related mortality were secondary endpoints. The search terms included “hematopoietic stem cell transplantation,” “antibacterial agent,” “antibiotic,” and “graft‐versus‐host disease” (Table S2).

Studies assessing the effect of antibiotic selection on overall survival, acute GVHD incidence, and GVHD‐related mortality were included. Review articles, case reports, and conference abstracts were excluded. Two researchers (H.I. and Y.O.) independently screened titles and abstracts, conducting full‐text reviews for shortlisted articles. Any disagreements regarding study selection were resolved in discussion with a third researcher (D.K.).

### 2.2 Data Extraction

Eligible study data were extracted into an Excel spreadsheet, including study details (author, year of publication, country, study design, and patient population), antibiotic regimens, underlying HSCT indications, and outcomes such as overall survival, acute GVHD incidence, and GVHD‐related mortality. When outcome data were presented in figures rather than text, we contacted authors for confirmation or estimated values using WebPlotDigitizer version 4 (https://automeris.io/WebPlotDigitizer/).

### 2.3 Risk of Bias Assessment

H.I. and Y.O. assessed bias risk using the Cochrane Risk of Bias tool 2.0 for randomized controlled trials (RCTs) and the Risk of Bias in Non‐Randomized Studies of Interventions (ROBINS‐I) for non‐RCTs.

### 2.4 Statistical Analysis

We conducted an exploratory meta‐analysis on overall survival, acute GVHD incidence, and GVHD‐related mortality to assess the clinical impact of antibiotics with activity against anaerobes and assess heterogeneity. When the frequencies of each event were unclear, approximate values were calculated from incidence rates. A binomial normal logistic regression model with maximum likelihood estimation was used to calculate risk ratios (RRs) and 95% confidence intervals (CIs). Heterogeneity was assessed using the *I* ^2^ test, with values > 50% indicating significant heterogeneity. A funnel plot for publication bias assessment was planned but not performed, as fewer than ten studies evaluated any single outcome [16]. All statistical analyses were conducted using EZR version 1.68 [17].

## 3 Results

**FIGURE 1:**
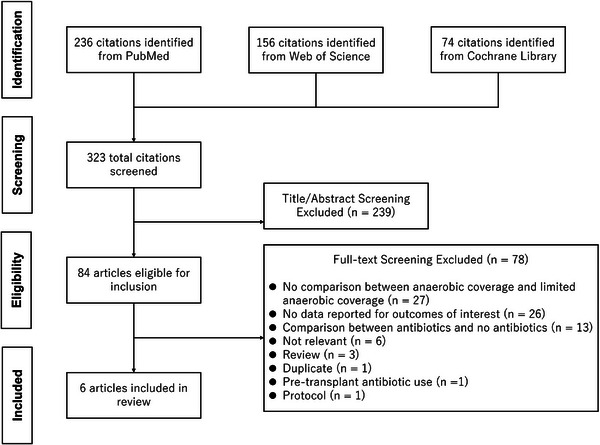
PRISMA flowchart illustrating the study selection process. PRISMA, Preferred Reporting Items for Systematic Reviews and Meta‐Analyses.

Of the 323 identified studies, six non‐RCTs comprising 2169 patients met the inclusion criteria, as illustrated in the PRISMA diagram (Figure 1) [8, 18–22]. No RCTs were eligible for inclusion. Five of the selected studies were conducted in the United States, while one was from Korea. Four were prospective cohort studies, and two were retrospective. The study by Jenq et al. compared GVHD‐related outcomes between groups with high and low intestinal microbiota diversity in patients who underwent allogeneic‐HSCT [18]; the study population consisted of two cohorts, which were defined based on differences in the sequencing platforms used for microbiota analysis. Jenq et al. presented individual patient data (e.g., antibiotics used, GVHD‐related outcomes) in the Supporting Information, and we considered this study eligible for inclusion in our review.

The median patient age ranged from 6–10 [20] to 51–54 years [22] across studies, with approximately 60% male participants (Table 1; Table S3). In all but one study (Simms‐Waldrip et al.), adult patients were included, over 60% of whom underwent HSCT for hematological malignancies. In contrast, the study by Simms‐Waldrip et al. sought to evaluate the impact of anti‐inflammatory Clostridia among pediatric patients [20]. Three studies compared outcomes between carbapenems or penicillins with beta‐lactamase inhibitors versus cephalosporins or aztreonam [8, 19, 21]. The remaining three studies examined the presence or absence of anaerobic coverage with additional agents such as metronidazole or clindamycin [18, 20, 22].

**TABLE 1:** Summary of studies included in the systematic review.

Author, year, country	Type of study	Antibiotics with anaerobic coverage	Antibiotics with limited anaerobic coverage	Indication for allogeneic HSCT	Age, median	Male, %	Follow‐up period
Jenq, 2015, the US	Prospective cohort study	Penicillins + BLI, carbapenems, MNZ, CLDM	FQs, cephalosporins, penicillins, AZT	Hematological malignancy (AML, MDS, ALL, etc.)	Cohort 1, 53 (range, 25–70) Cohort 2, 50 (range, 26–75)	Cohort 1, 62% Cohort 2, 69%	≤ 5 years
Shono, 2016, the US	Prospective cohort study	IPM, PIPC/TAZ	CFPM, AZT	Hematological malignancy (AML, MDS, ALL, etc.)	49 (range, 18–77)	60%	≤ 5 years
Simms‐Waldrip, 2017, the US	Prospective cohort study	Penicillins + BLI, carbapenems, MNZ, CLDM	FQs, cephalosporins	Sickle cell anemia, Hematological malignancy (AML, MDS, ALL, etc.)	6–10 (exact value unknown)	47%	≤ 100 days
Lee, 2019, Korea	Prospective cohort study	Carbapenems	CFPM	Hematological malignancy (AML, MDS, ALL, etc.)	AG, 45 (range, 18–65) LAG, 42 (range, 16–68)	AG, 68% LAG, 58%	≤ 1 year
Tanaka, 2020, the US	Retrospective cohort study	PIPC/TAZ, carbapenems	CFPM, CAZ, AZT	Both malignant (69%) and non‐malignant diseases (31%)	AG, 37 (IQR, 16–NA) LAG, 14 (IQR, 5–45)	AG, 57% LAG, 60%	≤ 1 year
Hanks, 2021, the US	Retrospective cohort study	PIPC/TAZ, carbapenems, MNZ	FQs, cephalosporins, AZT	Hematological malignancy (AML, MDS, ALL, etc.)	AG, 54 (range, 19–72) LAG, 51 (range, 17–72)	AG, 59% LAG, 53%	≤ 8 years

Abbreviations: AG, anaerobic coverage group; ALL, acute lymphoblastic leukemia; AML, acute myeloid leukemia; AZT, aztreonam; BLI, beta‐lactamase inhibitor; CAZ, ceftazidime; CFPM, cefepime; CLDM, clindamycin; FQ, fluoroquinolone; HSCT, hematopoietic stem cell transplantation; IPM, imipenem; IQR, interquartile range; LAG, limited anaerobic coverage group; MDS, myelodysplastic syndrome; MNZ, metronidazole; NA, not available; PIPC/TAZ, piperacillin‐tazobactam; the US, the United States.

### 3.1 Risk of Bias

**FIGURE 2:**
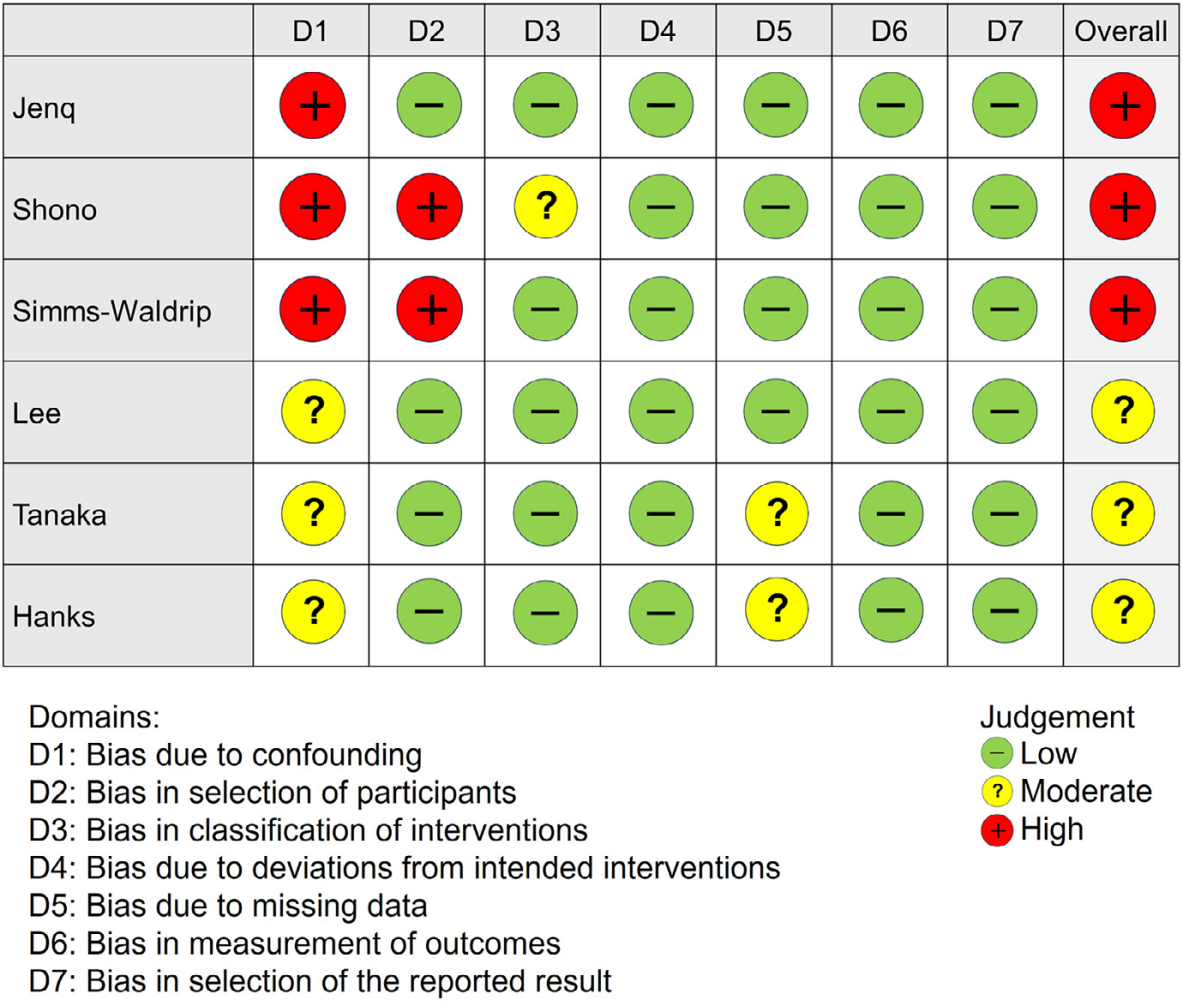
Risk of bias assessment for included studies.

Several studies did not adequately assess confounding factors such as patients' clinical status or comorbidities, and some had clinically and statistically significant baseline differences between groups, potentially introducing bias (Figure 2). In the study by Jenq et al., patient backgrounds by exposure group were unclear, increasing the risk of confounding bias [18]. Similarly, the study by Shono et al. lacked clarity in patient grouping, leading to both confounding and selection bias risks [19]. In Simms‐Waldrip et al., substantial imbalances in patient characteristics were present, and the exclusion of patients without appropriate fecal microbiota analysis further increased confounding and selection bias risks [20]. In the retrospective cohort studies by Tanaka et al. and Hanks et al., the approach to handling missing data was insufficiently detailed, raising concerns about data integrity [21, 22].

### 3.2 Outcomes

A meta‐analysis stratified by risk of bias was conducted to compare 1‐year overall survival, acute GVHD incidence, and GVHD‐related mortality between the anaerobic and limited anaerobic coverage groups (Table S4). A 5‐year overall survival meta‐analysis was not performed due to the limited number of studies with long‐term follow‐up data and the high risk of bias.

**FIGURE 3:**
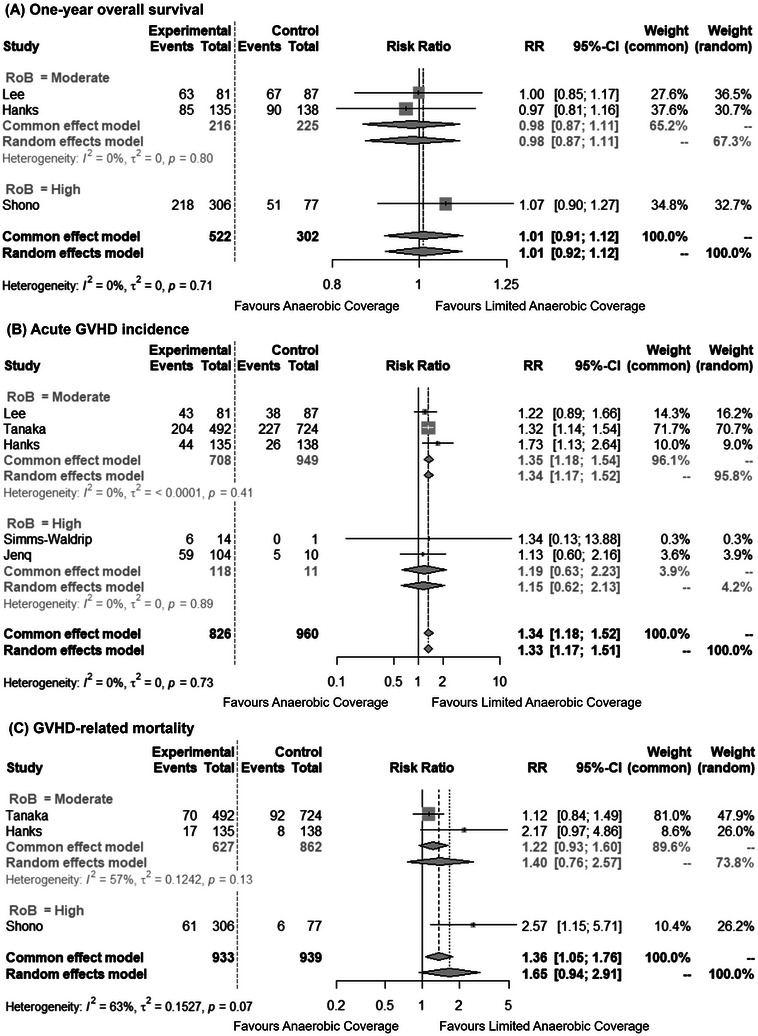
Forest plot of GVHD‐related outcomes: (A) 1‐year overall survival, (B) acute GVHD incidence, and (C) GVHD‐related mortality. CI, confidence interval; GVHD, graft‐versus‐host disease; RoB, risk of bias; RR, risk ratio.

Three studies (824 observations, 574 events) assessed 1‐year survival [8, 19, 22]. The random‐effects model yielded an RR of 1.01 (95% CI: 0.92–1.12), with no detected heterogeneity (*I* ^2^ = 0%) (Figure 3A).

Acute GVHD incidence was analyzed in five studies (1786 observations, 652 events) [8, 18, 20–22]. The random‐effects model estimated an RR of 1.33 (95% CI: 1.17–1.51), with no significant heterogeneity (I^2^ = 0%) (Figure 3B). Regarding intestinal GVHD incidence analyzed in three studies [8, 18, 22] and one additional study identified retrospectively by the third party [23], the random‐effects model estimated an RR of 1.51 (95% CI: 0.86–2.66), with moderate heterogeneity (*I* ^2^ = 60%) (Figure S1).

GVHD‐related mortality was analyzed in three studies (1872 observations, 254 events) [19, 21, 22]. The definition of GVHD‐related mortality was defined based on the Center for International Blood and Marrow Transplant Research algorithm [24] in the studies by Shono et al. and Hanks et al., while the definition in Tanaka et al. was based on the clinical judgment by the transplant physicians. The random‐effects model estimated an RR of 1.65 (95% CI: 0.94–2.91), with moderate heterogeneity (I^2^ = 63%) (Figure 3C).

## 4 Discussion

Antibiotics with anaerobic coverage did not improve 1‐year overall survival in patients who underwent allogeneic HSCT compared to those receiving antibiotics with limited anaerobic coverage. Instead, anaerobic coverage was significantly associated with higher acute GVHD incidence and showed a trend toward increased GVHD‐related mortality. While heterogeneity was minimal for 1‐year survival and acute GVHD incidence, it was moderate for GVHD‐related mortality. One possible explanation for the lack of survival benefit is that while broader antimicrobial coverage for FN may have helped manage infections, this potential advantage could have been offset by the increased risk and severity of GVHD, ultimately negating any survival benefit.

Acute GVHD incidence was higher in the anaerobic coverage group than in the limited anaerobic coverage group. This difference may be attributed to gut microbiota diversity, a key factor in GVHD protection [25]. A diverse gut microbiota enhances colonization resistance by inhibiting pathogen overgrowth through nutrient competition, niche exclusion, and antimicrobial production [26]. Furthermore, microbial diversity modulates immune responses, maintaining homeostasis and reducing excessive inflammation linked to GVHD [27, 28, 29]. Metabolically, it supports gut epithelial barrier integrity and immune regulation via short‐chain fatty acid production [30]. The resilience of a diverse microbiota ensures ecosystem stability, minimizing the effects of species loss [31]. Certain bacterial taxa, such as *Blautia* spp., are associated with reduced GVHD‐related inflammation [18]. Broad‐spectrum antibiotics with anaerobic coverage may disrupt this balance, weakening protective mechanisms and increasing GVHD susceptibility. In fact, even in studies including patients who underwent allogeneic HSCT, which were not included in our systematic review and meta‐analysis due to unclear information regarding patient exposure to antibiotics, the use of carbapenems was associated with the development of acute GVHD [32, 33]. On the other hand, another study including similar patients reported that exposure to fourth‐generation cephalosporins with limited anaerobic coverage was associated with the development of acute GVHD [34]; however, it should be noted that the control group in this study did not consist of patients who had received antibiotics with anaerobic coverage, but rather of those who had not been exposed to fourth‐generation cephalosporins. Our analytical results are consistent with the findings of these previous studies.

GVHD‐related mortality did not differ significantly between the anaerobic and limited anaerobic coverage groups. This may be explained by the relatively low severity of GVHD in the cohort, with a higher proportion of cases classified as Grade II, which has lower mortality rates than Grades III and IV [35, 36]. Hanks et al. reported that over half of acute GVHD cases in their study were Grade II, while Lee et al. found approximately 50% of cases in their cohort fell into this category. The relatively favorable prognosis of Grade II GVHD may have minimized differences in survival outcomes between groups despite the higher incidence of GVHD in the anaerobic coverage group.

A key strength of this study is its focus on the degree of anaerobic antibiotic coverage among patients who are post‐HSCT, which has been previously investigated in mouse experiments [23]. While previous research has explored anaerobic coverage in conditions such as pneumonia in non‐transplant patients [37, 38], few studies have compared post‐HSCT outcomes based on anaerobic coverage extent. Of note, a systematic review and meta‐analysis by Gavriilaki et al. has already examined the relationship between GVHD‐related outcomes and the presence or absence of antibiotic use in post‐HSCT patients [10]. However, while our systematic review and meta‐analysis assessed similar outcomes, it differs in that we focused on differences in the spectrum of antibiotics used. Our systematic review and meta‐analysis contribute essential evidence on the relationship between anaerobic bacterial presence in the gut microbiota and GVHD, offering valuable insights into this underexplored area.

This study has several limitations. First, only half of the included studies accounted for confounding factors (e.g., doner‐recipient relationship, myeloablative conditioning, GVHD prophylaxis regimen), while the remaining had a high risk of bias due to the lack of adjustment. To mitigate this, we performed a stratified analysis based on the risk of bias and confirmed that the meta‐analysis results remained consistent, even when high‐risk studies were excluded. Second, GVHD‐related mortality and overall survival were estimated using approximated RRs rather than raw data, as individual‐level data were unavailable in some studies. This necessitates a cautious interpretation of our findings. Third, the absence of RCTs in our analysis is a major limitation. Although our systematic search identified an RCT comparing rifaximin and ciprofloxacin for FN prevention in patients who are post‐HSCT [39], we excluded it because rifaximin is not a systemic antibiotic and does not meet the criteria for anaerobic coverage [40]. Third, most of the included studies focused on adults, making it unclear whether anaerobic coverage influences GVHD‐related outcomes in pediatric patients. Furthermore, while the types of antibiotics used were partially identified in the included studies, information on their dosage and duration of administration was unclear. These factors could have potentially affected GVHD‐related mortality.

In conclusion, this systematic review and meta‐analysis indicates that antibiotics with anaerobic coverage after allogeneic HSCT may be linked to a higher incidence of acute GVHD than those with limited anaerobic coverage. However, the absence of high‐quality studies, particularly RCTs, weakens the certainty of this finding. Further well‐designed research is essential to elucidate the impact of anaerobic antibiotic coverage on GVHD‐related outcomes and to inform clinical practice.

## Author Contributions

**Hiroshi Ito**: conceptualization, data curation, formal analysis, funding acquisition, investigation, methodology, project administration, resources, software, validation, visualization, writing – original draft. **Yui Okamura**: data curation, formal analysis, investigation, methodology, writing – review and editing. **Yuna Tomura**: data curation, formal analysis, investigation, methodology, resources, software, writing – review and editing. **Jura Oshida**: data curation, writing – review and editing. **Minori Fujita**: data curation, writing – review and editing. **Daiki Kobayashi**: conceptualization, formal analysis, supervision, writing – review and editing.

## Conflicts of Interest

The authors declare no conflicts of interest.

## Supporting information



Supporting Information

## Data Availability

Data sharing is not applicable to this article as no datasets were generated or analyzed during the current study.
